# Urbanization Reduces Phyllosphere Microbial Network Complexity and Species Richness of Camphor Trees

**DOI:** 10.3390/microorganisms11020233

**Published:** 2023-01-17

**Authors:** Yifang Zhang, Xiaomin Li, Lu Lu, Fuyi Huang, Hao Liu, Yu Zhang, Luhua Yang, Muhammad Usman, Shun Li

**Affiliations:** 1 College of Environmental Science and Engineering, China West Normal University, Nanchong 637009, China; 2 Key Laboratory of Urban Environment and Health, Ningbo Observation and Research Station, Institute of Urban Environment, Chinese Academy of Sciences, Xiamen 361021, China; 3 Department of Health and Environmental Sciences, Xi’an Jiaotong-Liverpool University, 111 Ren’ai Road, Suzhou 215123, China; 4 State Key Lab of Urban and Regional Ecology, Research Center for Eco-Environmental Sciences, Chinese Academy of Sciences, Beijing 100085, China; 5 PEIE Research Chair for the Development of Industrial Estates and Free Zones, Center for Environmental Studies and Research, Sultan Qaboos University, Al-Khoud, Muscat 123, Oman

**Keywords:** phyllosphere microbiota, epiphytes, co-occurrence networks, urban parks, suburban, microbial diversity

## Abstract

Studies on microbial communities associated with foliage in natural ecosystems have grown in number in recent years yet have rarely focused on urban ecosystems. With urbanization, phyllosphere microorganisms in the urban environment have come under pressures from increasing human activities. To explore the effects of urbanization on the phyllosphere microbial communities of urban ecosystems, we investigated the phyllosphere microbial structure and the diversity of camphor trees in eight parks along a suburban-to-urban gradient. The results showed that the number of ASVs (amplicon sequence variants), unique on the phyllosphere microbial communities of three different urbanization gradients, was 4.54 to 17.99 times higher than that of the shared ASVs. Specific microbial biomarkers were also found for leaf samples from each urbanization gradient. Moreover, significant differences (*R^2^* = 0.133, *p* = 0.005) were observed in the phyllosphere microbial structure among the three urbanization gradients. Alpha diversity and co-occurrence patterns of bacterial communities showed that urbanization can strongly reduce the complexity and species richness of the phyllosphere microbial network of camphor trees. Correlation analysis with environmental factors showed that leaf total carbon (C), nitrogen (N), and sulfur (S), as well as leaf C/N, soil pH, and artificial light intensity at night (ALIAN) were the important drivers in determining the divergence of phyllosphere microbial communities across the urbanization gradient. Together, we found that urbanization can affect the composition of the phyllosphere bacterial community of camphor trees, and that the interplay between human activities and plant microbial communities may contribute to shaping the urban microbiome.

## 1. Introduction

The phyllosphere, defined as the above-ground part of plants, has a total global foliar area of about 400 million km^2^ [1]. The phyllosphere thus can be a potential reservoir of microbial resources on Earth [2,3]. Plant leaves are the main organs for the transpiration and photosynthesis of green plants, and they are also inhabited with bacteria and fungi that can serve as natural openings connecting soil, water, and atmosphere. The phyllosphere microbiota has an important ecological service function in biogeochemical cycles and plant health [4,5]. Therefore, it is ecologically important to understand the microbial community compositions and biogeographic distribution patterns of the phyllosphere environment. With the development of molecular biology techniques, the phyllosphere microbiome has received increasing scientific attention in recent years. Microbial communities associated with plants in natural ecosystems have been studied extensively so far [6,7,8], but there exist limited data for urban ecosystems.

Due to rapid urbanization, more than 50% of the world’s population now lives in cities [9]. It is estimated that the urban population will increase by 2 to 4 billion from the current level in this century [10]. The benefits of urban vegetation on human health have been clearly demonstrated [11,12], while little is known about the potential role of human activities on urban plant microbiota. Recently, it has been shown that anthropogenic disturbances (such as land use practices [13], agronomic management systems [14,15], and artificial lights at night [16,17]) can affect the community composition of phyllosphere microbes. It is worth noting that the urbanization gradient is an important spatial proxy for studying how human activities affect the surroundings. To our knowledge, few studies have described changes in plant-associated microbiota along a suburban-to-urban gradient. How a plant microbiota responds to urbanization remains to be further elucidated. In this study, we focused on the phyllosphere bacterial communities of trees across the urbanization gradient and elucidated the similarities and differences in the phyllosphere microbial community structure of urban trees.

In many urban ecology studies, numerous parameters and methods have been used to estimate the intensity of human impacts on an area [18,19]. However, this hampered the comparison of experimental results from different studies. To standardize our work, we combined two quantifiable parameters, including population density (person km^−2^) and ALIAN, to characterize the urbanization gradient. Among them, population density is the number of people per unit of land area, and this is an important indicator of the distribution of population in a certain region [20]. Additionally, the Anthropocene has seen the amount of artificial light used at night increase significantly across globe. The ALIAN can indicate the degree of development of a region. Together, population density and ALIAN are considered to have the greatest impacts on urban ecosystems [21,22,23].

Camphor trees are members of a subtropical evergreen tree species and are often used as street trees in the southern cities of China [24,25]. Here, we collected 24 leaf samples from typical urban ecosystems in Ningbo city, China, and divided the sampling sites into urban, developing, and suburban areas based on the local urbanization gradient. This study aimed to (1) investigate the diversity and composition of the phyllosphere microbial community of camphor trees along a suburban-developing-urban gradient. Additionally, we sought to (2) explore the environmental factors affecting the regional distribution pattern of phyllosphere microbes across the urbanization gradient.

## 2. Materials and Methods

### 2.1. Sampling

In September 2020, camphor trees in eight urban parks (29.8434° N–29.9997° N, 121.4436° E–121.8392° E) (Figure S1) were selected for sampling according to the suburban-developing-urban gradient of Ningbo, China. As shown in Table S1, urbanization levels are vary, and the factors indicating the degree of urbanization include population density (person km^−2^) and ALIAN (W m^−2^ sr^−1^ µm^−1^). We determined the urbanization gradient based on the ALIAN and the regional population density. First, the ALIAN ranges of 1.19 × 10^−2^–3.57 × 10^−2^, 3.97 × 10^−4^–9.22 × 10^−4^, and 2.50 × 10^−7^–6.07 × 10^−5^ were classified as representing urban, developing, and suburban area, respectively. We then combined the population density data of the region and adjusted S3 (ca. 722 people km^−2^), the least densely populated part of the developing area, to a suburban area. The data of population density are adapted from Ningbo Statistical Yearbook (2021) (Table S1), and the data of ALIAN are obtained from the “Luojia-1” night light remote sensing satellite. The ALIAN of each sampling site is shown in Table S1 and Figure S2. Three randomly distributed camphor trees were chosen for sampling at each park. For each individual camphor tree, several intact and healthy leaves were collected at the same height with sterilized scissors and transferred to sterile bags (NASCO, Whirl-pak, Madison, WI, USA). The topsoil (0–15 cm in depth) near the tree was also collected and placed in a sterile bag. All leaf and soil samples were placed in a box with ice and immediately transported back to the lab for subsequent analyses.

### 2.2. DNA Extraction

The leaf samples were used for phyllosphere microbial DNA extraction. Around 7 g of leaves were weighed into a 250 mL sterilized conical flask, 200 mL of 0.01 M autoclaved PBS (phosphate buffer solution) was added, and then the mix was shaken at 160 rpm for 1.5 h under room temperature conditions [13,26]. The plant tissues were removed by filtration through a sterilized gauze. The rest solution was sterile-filtered using a 0.22 μm cellulose membrane and the membrane was stored at −20 °C until DNA extraction. DNA extraction was performed using the FastDNA^®^Spin Kit for soil (MP Biomedical, Santa Ana, CA, USA) kit. DNA samples were used for subsequent high-throughput sequencing, and the number of each sample is shown in the “Sample ID” column of Table S1. The remaining leaves were oven-dried at 65 °C to remove moisture and prepare for elemental analysis. Soil samples were air-dried and ground through a 2 mm sieve for use.

### 2.3. Leaf and Soil Physiochemical Properties

The leaf samples were dried in an oven at 65 °C to a constant weight; then, the water content was calculated based on the difference between wet weight and dry weight. The dried leaf samples were ground and crushed, and the total amounts of C, N, and S were determined using an elemental analyzer (Vario MAX CNS, Hanau, Germany). Soil NO_3_^−^, NO_2_^−^, and NH_4_^+^ concentrations were quantified using a flow injection analyzer (AutoAnalyzer 3, Bran Luebbe/SEAL Analytical, Norderstedt, Germany). Soil pH values were determined with a pH meter (Basic pH Meter PB-10, Goettingen, Germany) using the water-extraction redox potential method. The physicochemical properties of each sample are shown in Table S2.

### 2.4. High-Throughput Sequencing of 16S rRNA Genes

After DNA extraction, the V4 variable region of the bacterial 16S rRNA gene was amplified using a PCR (polymerase chain reaction) with forward primer 515F: 5′-GTGCCAGCMGCCGCGGTAA-3′ and reverse primer 806R: 5′-GGACTACHVGGGTWTCTAAT-3′, followed by sequencing on the Illumina MiSeq platform.

The downstream data, obtained from the Illumina MiSeq sequencing platform, were subjected to primer cleavage, quality filtering, denoising and merging of the raw sequence data and removal of chimeras to obtain the characteristic sequence ASVs using the DADA2 plug-in [27] in QIIME2 (2021.11). A given taxonomic annotation was compared to the Greengenes database (Release 13_8), and the ASVs of plant chloroplasts and mitochondria were removed from the species to obtain the taxonomic status of each sequence.

### 2.5. Statistical Analysis and Bioinformatics

All data analysis was carried out on the R software platform (version 4.2.0). Sample distributions and variances were assessed with the Shapiro–Wilk normality test and Levene test, respectively. The one-way ANOVA or Kruskal–Wallis tests were used to compare the differences in the relative abundance of microbiota, Shannon diversity index, Simpson diversity index, and species richness index of the phyllosphere microbial community of camphor trees at different urbanization levels. To analyze the statistical significance of the standard deviations of normal distributions between more than two groups, ordinary one-way ANOVA and Duncan’s post hoc test were used. For multiple comparisons where at least one group was not normally distributed or had the same standard deviation, Kruskal–Wallis test and Dunn’s post hoc test were used. In all cases, statistical significance was assessed with 95% confidence intervals, so that *p* < 0.05 was considered significant.

The “Venn diagram” package was used to create a Venn diagram. Based on the Bray–Curtis distance algorithm, the “vegan” package was run to identify distribution patterns of phyllosphere microbial community composition across urbanization gradient. Based on the same distance matrix, PERMANOVA was performed using the “adnois” function of the “vegan” package to measure the significant differences of the phyllosphere microbial community between different urbanization gradients. The number of permutations was 999. LEfSe (Linear discriminant analysis Effect Size) analysis was performed using the “microbiomeMarker” package, and the LDA = 3 discriminant score was used as a threshold to classify various biomarkers. In view of the differences in sequencing depth between samples, 31,399 sequences were rarefied and analyzed with the “iNEXT” package when calculating alpha diversity. The network analysis was visualized in Gephi (version 0.9.2) using the routine CoNet [28]. Before constructing the network, the ASVs were removed that occur for less than 70% of all samples. Spearman [29] correlation coefficient between pairwise ASVs was calculated in R using the relative abundance of ASVs, and only robust (Spearman’s r > 0.6 or r < 0.6) and statistically significant (*p* < 0.05) network nodes were kept. To analyze the network topology, the “igraph” package is used to calculate the basic network properties. The network complexity is defined according to the results of previous studies [30,31]. Core microbial species were identified by within-module connectivity (Zi) and among-module connectivity (Pi) assessments [32]. RDA (Redundancy analysis) of the effect of environmental factors on microbial community structure was performed using the “vegan” package, and then the “rdacca.hp” package was used to decompose the explanation rate of each environmental factor by using the hierarchical partitioning theory. All data obtained above were visualized using the “ggplot2” package.

## 3. Results

### 3.1. Phyllosphere Microbial Community Composition of Camphor Trees

**Figure 1:**
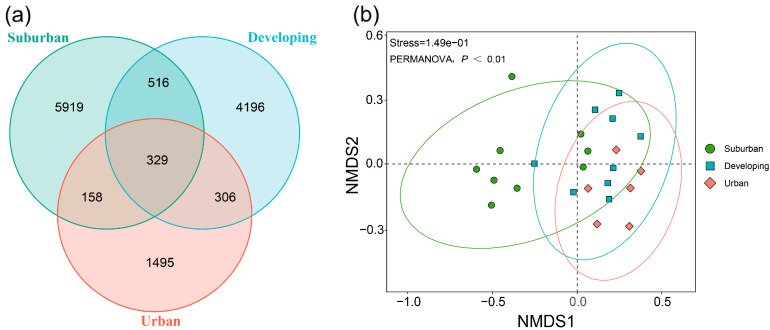
Changes in microbial community composition and diversity along the urbanization gradient. Venn diagram (**a**) and NMDS (Non-metric multidimensional scaling) analysis (**b**) of phyllosphere microbiota of camphor trees with different urbanization levels. Suburban, developing, and urban stand for the corresponding urbanization level for sampling.

A total of 170,1603 ASVs was obtained by high-throughput sequencing of the V4 region of the 16S rRNA gene, with the lowest for sample U2-2 (48,707 ASVs) and the highest for sample S1-3 (91,859 ASVs) (Table S1). Only 329 ASVs were shared among the three urbanization-level phyllosphere samples, with the suburban region having the highest number of unique microbial species (5919 ASVs), followed by the developing region (4196 ASVs) (Figure 1a). In other words, the number of ASVs unique to the leaf samples of the three urbanization levels was 4.54–17.99 times higher than the number of shared ones, indicating that the species composition of the phyllosphere microbial community of camphor trees differs greatly across the urbanization gradient.

NMDS (Non-metric multidimensional scaling) analysis, based on PERMANOVA of the Bray–Curtis distance community structure, was used to assess camphor leaf microbiota along the urbanization gradient (Figure 1b). We found that the bacterial community structure of camphor phyllosphere microbiota showed significant differences with the urbanization gradient (R^2^ = 0.13, *p* = 0.005). Further, we performed paired adonis similarity analyses on phyllosphere microbiota, and found that differences in phyllosphere microbial community structure existed mainly between suburban and urban camphor leaves (R^2^ = 0.25, *p* = 0.018). The results of PERMANOVA analysis based on Bray–Curtis distance are shown in Table S3. Namely, there was significant difference in the phyllosphere microbial structure between suburban and developing camphor leaves (R^2^ = 0.19, *p* = 0.039), while there was not significant difference between the developing and the urban camphor leaves (R^2^ = 0.07, *p* = 0.481).

**Figure 2:**
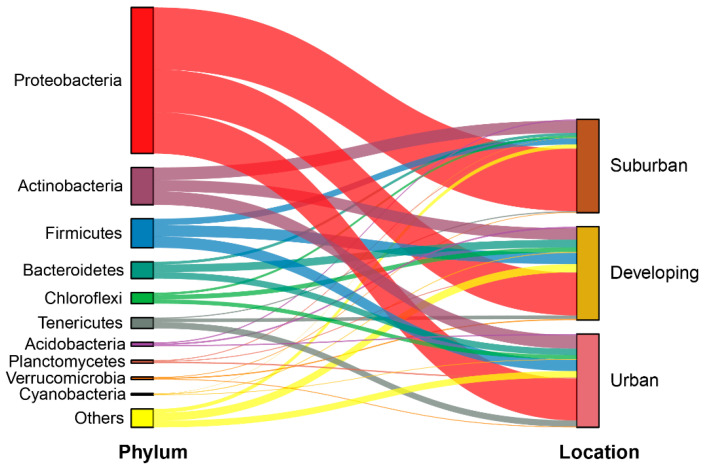
Relative abundance of top 10 phyla of the phyllosphere microbiota along the urbanization gradient. Only the top 10 species at the phylum taxonomic level are shown, and remainder of species are referred to as others. Suburban, developing, and urban stand for the corresponding urbanization level for sampling.

All sequences identified as 16S rRNA in the sample sites were classified into 54 phyla, 166 classes, 326 orders, 524 families, and 961 genera. Overall, the average relative abundance of Proteobacteria, Actinomycetes, Firmicutes, and Bacteroidetes was 53.15%, 13.16%, 10.15%, and 5.90%, respectively. These accounted for 82.36% of the total sequences and were the dominant species of phylum (Figure 2).

### 3.2. Species Composition Variability and Biomarkers of Camphor Phyllosphere Microbiota

**Figure 3:**
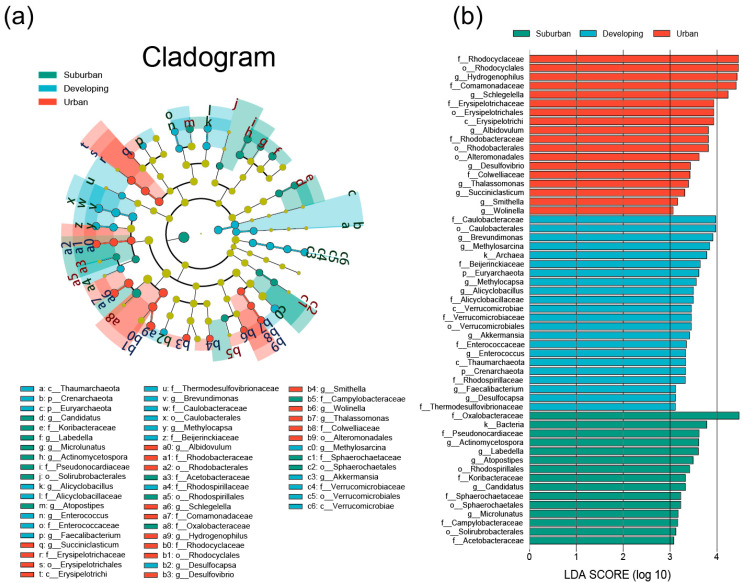
LEfSe(Linear discriminant analysis Effect Size) analysis of phyllosphere microbiota along the urbanization gradient. Evolutionary branching diagram (**a**) and LDA scores (**b**) for biomarkers of camphor trees with different urbanization levels. Suburban, developing, and urban stand for the corresponding biomarkers of different urbanization levels. The yellow nodes represent microbiota that showed no significant difference across the urbanization gradient. LDA SCORE (log10) indicates linear discriminant analysis score.

To determine the main differences in the composition of phyllosphere microbiota (from phylum to genus level) across the urbanization gradient, we performed LEfSe analysis and observed 55 different abundant taxa (LDA value > 3.0). The results are shown in Figure 3 and Table S4. We found that the phyllosphere microbiota in the suburban, developing, and urban areas have their unique biomarkers, that is, specific abundant species. There were 21, 18, and 9 biomarkers at the genus, family, and order levels, respectively. At the phylum level, the phyllosphere microbiota of suburban camphor trees were dominated by *Actinobacteria* and *Proteobacteria*. Besides, *Proteobacteria*, *Firmicutes*, and *Verrucomicrobia* were significantly enriched on the camphor phyllosphere in the developing area. For the urban phyllosphere microbiota, all 18 abundant species belonged to *Proteobacteria* and *Firmicutes*. The statistical *p* values of the relative abundance of all microbiota with different urbanization levels are shown in Table S4.

As shown in Figure 3, *Atopostipes* (*p* = 0.0021, LDA score = 3.49) and *Acetobacteraceae* (*p* = 0.0315, LDA score = 3.08) were significantly enriched on the leaves of suburban camphor trees with an optimum growth temperature of 28~30 °C [33] and 28~33 °C [34], respectively, and were not heat tolerant. In contrast, *Hydrogenophilus* (*p* = 0.0021, LDA score = 3.49), *Schlegelella* (*p* = 0.0345, LDA score = 4.24), and *Albidovulum* (*p* = 0.0424, LDA score = 3.81), which were markedly enriched in urban areas, were thermophilic bacteria with optimum growth temperatures of 50~52 °C [35], 50 °C [36], and 50 °C [37], respectively. Only in suburban areas can *Oxalobacteraceae* (*p* = 0.0399, LDA score = 4.47) be markedly enriched, and this family includes nitrogen-fixing microbiota [38]. Notably, we found that *Erysipelotrichales* (*p* = 0.0213, LDA score = 3.93) and *Erysipelotrichaceae* (*p* = 0.0213, LDA score = 3.93), which are significantly enriched in urban areas, are pathogenic to mammals and birds [39,40].

### 3.3. Phyllosphere Microbial Community Diversity and Network Complexity

**Figure 4:**
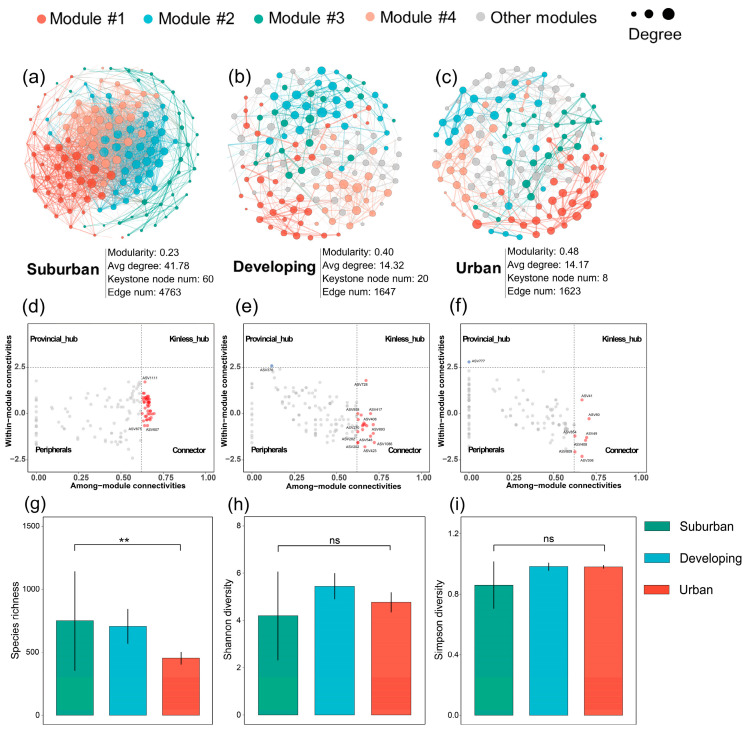
Co-occurrence networks (**a**–**c**), node network roles (**d**–**f**), and alpha diversity indexers (**g**–**i**) of phyllosphere microbiota of camphor trees along the urbanization gradient. Suburban, developing, and urban stand for the corresponding urbanization level for sampling. Avg degree—average degree; Keystone node num—keystone node numbers; Edge num—edge numbers. Threshold value for node network roles: kinless hubs (*z* score > 2.5; *c* score > 0.62), provincial hubs (*z* score > 2.5; *c* score ≤ 0.62), connectors (*z* score ≤ 2.5; *c* score > 0.62), and peripherals (*z* score ≤ 2.5; *c* score ≤ 0.62) were defined according to their within-module degree (*z* score) and among-module degree (*c* score). An asterisk denotes a significant statistical difference between treatments (**, *p* < 0.01), and ns indicates that the statistical difference is not significant.

To further evaluate the effects of urbanization on the phyllosphere microbiota of camphor trees, we assessed the alpha diversity and symbiotic patterns of bacterial communities (Figure 4). The degree of the bacterial co-occurrence network conformed to a power–law distribution (Figure S3). We calculated species diversity with Shannon diversity and Simpson diversity indexes, and estimated species richness using the species richness index. Our results show that urbanization can strikingly reduce the network complexity (the higher the average degree, the higher the network complexity [41]) and species richness of phyllosphere microbiota. Compared to the suburban area, species richness decreased significantly with urbanization (*p* = 0.0064). However, there was no significant difference in species diversity of camphor trees across all urbanization levels (*p* = 0.1529 for Shannon diversity and *p* = 0.1107 for Simpson diversity), and the α diversity indexes are shown in Table 1. The average degree indicating bacterial network complexity gradually decreased from suburban (41.78) to developing (14.32) and to urban areas (14.17), with the lowest bacterial richness and network complexity seen for the phyllosphere microbiota of urban camphor trees (Figure 4 and Table S6).

Recently, growing studies have shown that there are keystone taxa in microbial communities whose effects on community composition and function are independent of their abundance [42,43,44]. We found the number of keystone species gradually decreased from suburban (43) to developing (20) and to urban areas (8) (Figure 4d–f). The keystone taxa are listed in Table S5. Additionally, urbanization significantly altered the interaction pattern of the phyllosphere microbiota. The developing and urban areas had relative higher modularity and negative correlation edges (Figure 4a–c; Table S6), and the negative correlations increased with increasing urbanization levels (the ratio of P/N, from 12.12 to 1.39 and to 1.16). In particular, we found that the interactions associated with keystone species changed from being mostly positive to mostly negative along the suburban-to-urban gradient (Figure S4).

### 3.4. Correlations between Environmental Factors and Microbial Community Structure

**Figure 5:**
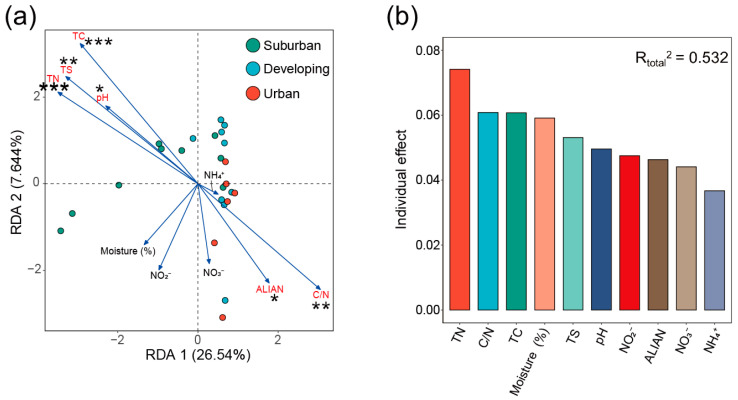
RDA (Redundancy analysis) of environmental factors and microbial communities (**a**), and the effect of each environmental factor on microbial community structure (**b**). Suburban, developing, and urban stand for the corresponding urbanization level for sampling. The dots in different colors represent the microbial samples along the urbanization gradient, and the length of the arrows of the physicochemical factors can represent the influence magnitude of the physicochemical factors. ALIAN—artificial light intensity at night. Moisture (%)—Water content (%) of camphor leaves with different urbanization levels. R_total_^2^—Explanation rate of the full model (i.e., including all environmental factors). An asterisk denotes a significant statistical difference between treatments (*, *p* < 0.05; **, *p* < 0.01, ***, *p* < 0.001), and ns indicates that the statistical difference is not significant.

The results showed that some environmental factors varied across the urbanization gradient and may have contributed to altering the structure of the camphor tree bacterial community (Table S2, Figure 5). The soil pH was significantly lower in the developing and urban areas than in the suburban areas (*p* = 0.001), and leaf C, N, and S of camphor leaves decreased markedly with urbanization (*p* = 0.0003 for TC, *p* = 2.78 × 10^−5^ for TN, and *p* = 0.0002 for TS). Additionally, ALIAN were significantly enhanced (*p* = 1.09 × 10^−4^) in the urban area, while other parameters showed no significant difference along the suburban-to-urban gradient.

**Figure 6:**
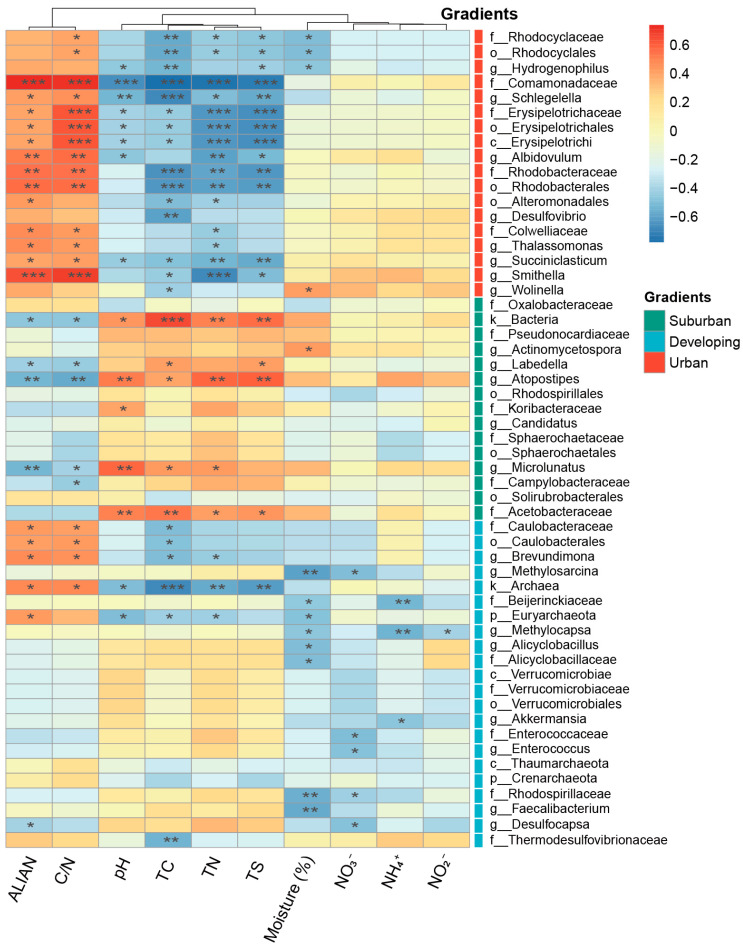
Spearman correlation analysis of biomarkers and environmental factors. Suburban, developing, and urban stand for the corresponding biomarkers of different urbanization levels. An asterisk denotes a significant statistical difference between treatments (*, *p* < 0.05; **, *p* < 0.01; ***, *p* < 0.001), and the blank indicates that the statistical difference is not significant. ALIAN—artificial light intensity at night. Moisture (%)—water content (%) of camphor leaves along the urbanization gradient.

We performed RDA of the relative abundance of keystone species, identified by network analysis and 10 environmental factors (Figure 5a). The RDA results showed that leaf C, N, S, C/N, soil pH, and ALIAN had significant impacts on the structure of the camphor phyllosphere keystone microbiota (Figure 5a, Table 2). To further evaluate the effect of each environmental factor on the microbial community structure, we used the “rdacca.hp” package to distinguish the explanation rate of each explanatory variable in the RDA by using the hierarchical partitioning theory (Figure 5b, Table 2). We found that the highest explanation rates were leaf N, C/N, C, moisture content (%), and leaf S, indicating that leaf physicochemical properties could be important drivers shaping in the phyllosphere microbiota of camphor leaves. To further explore the relationship between environmental factors and microbial community composition, Spearman correlation analysis was performed using the biomarkers screened by LEfSe analysis and environmental factors (Figure 6). The results further support our conclusions. They showed that suburban biomarkers were positively correlated with leaf C, N, S, and soil pH, and negatively correlated with ALIAN and leaf C/N. In contrast, for leaf physicochemical properties, pH and ALIAN, we found opposite results for Spearman correlation analysis of the phyllosphere biomarkers of urban camphor trees compared to the suburban camphor trees.

## 4. Discussion

### 4.1. Urbanization Changed the Composition of Camphor Phyllosphere Microbiota

The urbanization significantly altered the phyllosphere microbial community and selected different microbial taxa. Three thermophilic bacteria, *Hydrogenophilus*, *Schlegelella*, and *Albidovulum*, were markedly enriched on the urban camphor phyllosphere microbial community. It is known that extreme conditions of the leaf surface (e.g., temperature, humidity, and solar radiation) could have a large impact on the phyllosphere microbiota [45], leading to the selection of certain microbiota residing in the phyllosphere. For instance, Bernard et al. [46] found that the temperature of the leaf surface can directly affect the phylogenetic development of phyllosphere microbiota. Moreover, Godon et al. [47] found that the leaf surface can be heated by the sun and absorbed a large amount of energy from solar radiation, thereby resulting in the phyllosphere being an important habitat for thermophilic bacteria. Therefore, we speculate that the three thermophilic bacteria which were enriched in urban areas may be related to the urban heat island effect. The potential impact of climate warming on the abundance and composition of phyllosphere microbiota has only attracted attention in recent years, and our findings may contribute to a better understanding of how human activities affect the phyllosphere microbiota of urban plants. Urban phyllosphere microbiota are under increasing pressure due to intensified climate extremes, especially warming and drought. These stresses may lead to an unstable state of microbial communities, causing such phenomena as a reduction in beneficial taxa [48]. As an illustrative example, we found that the nitrogen-fixing microbiota can only be enriched in suburban areas. Atmospheric N_2_, fixed by phyllosphere nitrogen-fixing microbiota, can be directly taken up by leaves or washed by rain and then acquired by roots, thereby supporting plant growth [49,50]. Notably, the pathogenic bacteria *Erysipelotrichales* and *Erysipelotrichaceae* are biomarkers in urban areas that may suggest that human activities can potentially increase the spread of pathogens in urban ecosystems. In contrast, *Atopostipes*, markedly enriched in suburban areas, were isolated from underground pig-manure storage pits [33] and widely colonized the gastrointestinal tract of animals such as pigs and chickens [51,52,53]. The application of farmyard fertilizer in suburban areas may lead to the enrichment of *Atopostipes*. Interestingly, *Rhodobacterales* (*p* = 0.0177, LDA score = 3.81) and *Rhodobacteraceae* (*p* = 0.0247, LDA score = 3.81) were significantly enriched on the urban camphor leaves and mainly include photoautotrophic and chemoautotrophic photosynthetic bacteria [54]. Additionally, *Albidovulum* was also enriched in the phyllosphere of urban camphor trees; which has previously been shown to be tightly linked to species of the photosynthetic genus, such as *Rhodovulum* [37]. Therefore, we assume that ALIAN may also affect the phyllosphere microbial community. However, more direct evidence is needed to support this speculation.

### 4.2. Urbanization Reduced the Complexity and Species Richness of Phyllosphere Microbial Networks of Camphor Trees

Our results showed that urbanization can reduce the network complexity and species richness of phyllosphere microbiota. It has been shown that with the intensification of agricultural land and urbanization, biodiversity is under increasing pressure from human activities (such as climate change and pollution [55] and global biodiversity is declining [56]. This may explain the significantly lower phyllosphere microbial richness of urban camphor trees than that of suburban camphor trees, and may partly explain the lower complexity of the phyllosphere bacterial networks of urban camphor trees.

Network analysis showed that negative correlations in the phyllosphere bacterial network of camphor trees increased with the urbanization level. With increased human activities, the Earth is undergoing dramatic changes such as environmental pollution, frequent climate extremes, global warming, and increased risk of drought [57]. For example, Annamalai [58] et al. showed that urban expansion and human activities are important sources of various air pollutants. Compared to non-urban trees, these chemicals, as well as having additional macro and trace elements, are being enriched in the leaves [59,60]. As such, they may potentially affect the leaf surface environment. These changes may intensify microbial competition for nutrient and growth resources under pollution stress, thereby promoting negative symbiotic patterns among related microbes. Additionally, keystone taxa changed with urbanization. *Thermogemmatisporaceae*, *Lachnospiraceae*, *Comamonadaceae*, and *Pseudomonadaceae* were observed only in keystone species of the suburban bacterial network, and such microbiota have high capacity to degrade macromolecular organic matter, such as cellulose [61], starch [62,63], organic acids [64], and proteins [65]). Moreover, the aerobic nitrifier *Nitrobacteria* was only observed in keystone taxa of the urban bacterial network. As the main alkaline gas in the atmosphere, ammonia is not only a main cause for PM_2.5_ pollution, but is also an important contributor to atmospheric nitrogen deposition [66]. It is known that human activities have a large impact on plants; in turn, plant microbiota can remediate air pollutants [67] (such as degrading pollutants deposited on leaf surfaces) and affect human health [68]. This result suggests that urbanization could increase atmospheric nitrogen inputs (in the form of NH_x_ and NO_y_) onto the phyllosphere of urban trees, and will likely drive a shift in keystone species from organic matter-degrading microbiota (e.g., *Thermogemmatisporaceae*, *Lachnospiraceae*, and *Pseudomonadaceae*) to ammonia-nitrogen metabolizing microbiota (e.g., *Nitrobacteria*).

The keystone taxa contained abundant functional microbiota related to plant growth such as nitrogen-fixing bacteria, photosynthetic bacteria, and phosphorus-dissolving bacteria, while contained fewer potential plant pathogens. We observed *Methylobacteriaceae*, which is a family of the order *Rhizobiales* in keystone taxa of phyllosphere microbial network of suburban, developing and urban camphor trees [69]. We also observed *Actinomycetales* that can parasitize the roots of alder and other plants to form root nodules and perform nitrogen fixation in keystone taxa of the phyllosphere microbial network in suburban and developing areas [70]. They could play an important role in the nitrogen cycle of urban ecosystems. Moreover, we observed *Chloroflexi* that can produce energy through photosynthesis in keystone taxa of the phyllosphere microbial network in suburban and developing camphor trees [71]. Compared with other microbiota, keystone taxa also include functional microbiota relevant to redox reactions and elemental cycling. For example, we observed *Methylosarcina* which is a biomarker group for methane oxidation [72] in keystone taxa of the bacterial network of suburban and developing camphor trees. We observed *Massilia* among keystone taxa of suburban camphor phyllosphere microbial network, and members of this microbe often exhibit multiple physiological activities. For instance, *Massilia tieshanensis TS3T* can tolerate various heavy metals such as As^3+^, Cu^2+^, Zn^2+^, Ni^2+^, and Cd^2+^ [73], and can degrade polycyclic aromatic hydrocarbons and chloroacetamide herbicides [74,75], while also solubilizing phosphorous [76]. The keystone taxa in the suburban phyllosphere microbial network have diverse metabolic pathways and could adapt to multiple environmental conditions, this can be direct evidence for higher network complexity and indirect evidence for higher species richness. Growing evidence showed the profound and harmful impacts of human activities on plant health and ecosystem functions. Thus, utilizing the ecological service function provided by the phyllosphere microbiota to enhance plant growth and adaption to global changes is urgent and considered as a sustainable approach [77,78,79]. Our results provide a basis for the interaction between urbanization and the phyllosphere microbial community, and may have important implications for better understanding the microbial ecology of urban ecosystems.

### 4.3. The Phyllosphere Microbial Community of Camphor Trees Are Related to Environmental Factors

Some environmental factors varied across the urbanization gradient and affected the bacterial community structure of camphor tree phyllosphere (Figure 5). Soil pH is known to be sensitive to human activities (e.g., land use practices and traffic pollution) [80,81]. Although developing- and urban-area parks are not industrial sites, the dense population and constant passage of traffic vehicles may be major factors affecting soil chemistry. Moreover, changes in soil physical properties with urbanization may be important factors affecting the growth of urban plants. Human activities (e.g., artificial compaction with construction wastes) may alter the physicochemical properties of urban soils, resulting in a decrease in soil nutrients [82]. This may minimize the function of soils to maintain plant growth and decrease the nutrient supply to plants. This may lead to a remarkable decrease in the leaf C, N, and S of urban camphor leaves. Additionally, urban areas have increasingly artificial lights under rapid urbanization conditions [83]. Therefore, urban camphor trees are exposed to a significantly higher artificial light intensity than that suburban camphor trees face.

RDA of the relative abundance of keystone microbes and 10 environmental factors showed that the order of explanation rate of each explanatory variable from high to low were leaf N, C/N, C, moisture content (%), leaf S, soil pH, soil NO_2_^−^, ALIAN, soil NO_3_^−^, and soil NH_4_^+^. This is consistent with results of previous studies. These reported that leaf physicochemical properties were important drivers of the phyllosphere microbial community of camphor trees, and that these essential nutrients are available resources for microbiota living on the phyllosphere, thus influencing the phyllosphere microbial community structure [84,85,86]. In addition, soil pH can affect the elemental content of leaves by influencing the nutrient transport from the soil to the plant. For example, Zhao et al. [87] found that soil pH may alter the effective nutrients in the soil, leading to an imbalance of elements in plants. Moisture is also an important factor. For example, Morris [88] et al. showed that the increase in effective water in the phyllosphere environment can reduce the physiological stress of microbiota and may affect the physiological function and structure of the microbial community. Additionally, water content can influence the microbial community structure by affecting the mobility and availability of nutrients required for microbiota [89]. Moreover, ALIAN could also explain part of the variation in the phyllosphere microbial community. Some photosynthetic bacteria can perform photosynthesis under artificial lights at night, a process which may lead to changes in the microbial community. As reported by Alsanius et al. [90], phyllosphere microbiota benefit not only by exchanging compounds with host plants, but also by light, and that artificial lighting may shape the phyllosphere microbiota community structure of greenhouse-grown sunflowers. In contrast, artificial lights at night also have a negative effect on the growth of microbiota with circadian rhythms. It has been shown that many biological traits of plants are related to external light and internal biological clocks, exhibiting corresponding behaviors throughout the day [91]. In addition to plants, their phyllosphere microbiota may also be affected by light and biological clocks [92]. Artificial lights at night in urban ecosystems may disturb the circadian rhythms of phyllosphere microbiota and thus inhibit their growth. The results of ALIAN Spearman correlation analysis further support the speculation mentioned in the RDA. Artificial lights at night is a double-edged sword, negatively affecting the growth of microbiota through disturbing circadian rhythms, while positively influencing the growth of taxa that benefit from artificial lights at night. Notably, according to the spearman correlation heatmap, suburban biomarkers were positively correlated with leaf C, N, and S, while urban biomarkers were negatively correlated with leaf C, N, and S. We speculate that some photosynthetic bacteria in urban phyllosphere microbiota can perform photosynthesis to some extent under artificial lights, thus inducing more oxygen and DOC (dissolved organic carbon ) increase. These changes may partly alleviated the dilemma of nutrient limitation in the phyllosphere. In general, the measured 10 environmental factors explained a total of 53.20% of the variation in the phyllosphere microbial community of camphor trees in urban ecosystems (Figure 5b), our results suggest that future relevant studies should explore other determinants and random factors that may influence the phyllosphere microbial community structure.

## 5. Conclusions

In this study, we explored the composition and diversity of phyllosphere microbial communities of camphor trees from eight parks along a suburban-developing-urban gradient, and revealed the spatial distribution patterns of phyllosphere microbes with urbanization. The results showed that the composition and spatial distribution of phyllosphere microbial community of camphor trees were different with the urbanization gradient. Differences in phyllosphere microbial community structure mainly existed between urban and suburban camphor trees. Urbanization could reduce the complexity and species richness of the phyllosphere microbial network. Leaf C, N, S, C/N, soil pH, and ALIAN were the environmental factors that may play a major role in changing camphor trees’ phyllosphere microbial community. In conclusion, urbanization can markedly affect the diversity and spatial distribution patterns of phyllosphere microbial communities of camphor trees. This improves our understanding of the urban tree microbiome. However, a case study that based its conclusions on only one urbanization gradient could be far from sufficient, and future investigations at larger scales are needed. We propose that more efforts are needed in the future to maintain plant-associated microbial diversity in urban ecosystems and to clarify which plant species are most effective in pollutant degradation and a reduction in the spread of disease-causing microbiota. Moreover, the beneficial microbiota could be screened for air remediation and plant productivity. In short, the utilization of phyllosphere microbial community should aim to maximize their ecosystem service function, thereby contributing to sustain “one health” of our planet.

## Figures and Tables

**Table 1:** The alpha diversity indexes of leaf phyllosphere microbiota of different urbanization levels.

Urbanization Gradient	Species Richness	Shannon Diversity	Simpson Diversity
Suburban	888.67 ± 556.78 a	4.2 ± 1.86 a	0.86 ± 0.15 a
Developing	704.22 ± 136.74 ab	5.44 ± 0.55 a	0.98 ± 0.03 a
Urban	454.67 ± 47.84 b	4.77 ± 0.42 a	0.98 ± 0.01 a

Suburban, developing, and urban stand for the corresponding urbanization level of a sample. Values are mean ± standard deviation, and different lowercase letters after the same column of figures indicate significant differences.

**Table 2:** RDA (Redundancy analysis) of leaf phyllosphere microbiota of different urbanization levels.

	Physicochemical Parameter	*r* ^2^	*p* Value	Individual Effect
Leaf	C (%)	0.6061	0.001	0.0607
	N (%)	0.5101	0.001	0.0741
	S (%)	0.5293	0.002	0.0531
	C/N	0.4717	0.001	0.0608
	Moisture content (%)	0.1121	0.278	0.0591
Soil	NH_4_^+^	0.0077	0.940	0.0367
	NO_2_^−^	0.1260	0.229	0.0475
	NO_3_^−^	0.0936	0.326	0.0441
	pH	0.2588	0.047	0.0496
Light	ALIAN	0.2674	0.043	0.0463

*r*^2^—the axis radius of each environmental factor in the RDA diagram. Individual effect—the explanation rate of each variable in the RDA. ALIAN—artificial light intensity at night.

## Data Availability

Raw sequence data, metadata, and bioinformatic pipeline used in Qiime2 are available at: https://www.ncbi.nlm.nih.gov/bioproject/PRJNA919006.

## Supplementary Materials

The following supporting information can be downloaded at: https://www.mdpi.com/article/10.3390/microorganisms11020233/s1, Figure S1: Map of sampling sites for the eight investigated parks along the urbanization gradient; Figure S2: Artificial light intensity at night of 24 sampling sites in 8 parks; Figure S3: The network degree distribution patterns of phyllosphere microbiota of camphor trees with different urbanization gradients; Figure S4: Co-occurrence networks of phyllosphere microbiota of camphor trees with different urbanization gradients; Table S1: Geographical information of sampling sites from different city parks; Table S2: The physicochemical properties of the leaf and soil samples plus the light intensity; Table S3: PERMANOVA analysis of Bray-Curtis dissimilarities of phyllosphere microbiota of camphor trees with different urbanization gradients; Table S4: Biomarkers screened by LEfSe analysis of phyllosphere microbiota of camphor trees with different urbanization gradients; Table S5: The keystone taxa of phyllosphere bacterial networks of camphor trees with different urbanization gradients; Table S6: Bacterial co-occurrence network of phyllosphere microbiota samples under different urbanization gradients.
