# Supplementary material for: Urbanization Reduces Phyllosphere Microbial Network Complexity and Species Richness of Camphor Trees
Source: Microorganisms. 2023 Jan 17;11(2):233. doi: 10.3390/microorganisms11020233 (PMC9966171; doi:10.3390/microorganisms11020233)
Supplement: Supplementary file 1 [file microorganisms-11-00233-s001.zip › microorganisms-2119197-supplementary.pdf]

Supporting Information

Urbanization reduces microbial network complexity and species richness of camphor trees

Yifang Zhang [1,2], Xiaomin Li [2], Lu Lu [1, *], Fuyi Huang [2], Hao Liu [3], Yu Zhang [4], Luhua Yang [2], Muhammad Usman [5], Shun Li [2, *]

[1] College of Environmental Science and Engineering, China West Normal University, Nanchong 637009, China.

[2] Key Laboratory of Urban Environment and Health, Ningbo Observation and Research Station, Institute of Urban Environment, Chinese Academy of Sciences, Xiamen 361021, China.

[3] Department of Health and Environmental Sciences, Xi'an Jiaotong-Liverpool University, 111 Ren'ai Road, Suzhou, Jiangsu 215123, China.

[4] State Key Lab of Urban and Regional Ecology, Research Center for Eco-Environmental Sciences, Chinese Academy of Sciences, Beijing 10085, China.

[5] PEIE Research Chair for the Development of Industrial Estates and Free Zones, Center for Environmental Studies and Research, Sultan Qaboos University, Al-Khoud 123, Muscat, Oman.

[*] Correspondence: llu327@cwnu.edu.cn (L. Lu); snli@iue.ac.cn (S. Li)

Table S1. Geographical information of sampling sites from different city parks.

Sample ID	Latitude and longitude (°)	Population density (person km^{-2})	ALIAN ($W\ m^{-2}\ sr^{-1}\ \mu m^{-1}$)	Urbanization level
S1-1	29.9985°N， 121.4570°E	1283.13	2.50×10^{-7}	Suburban
S1-2	29.9997°N， 121.4546°E	1283.13	1.68×10^{-6}	Suburban
S1-3	29.9985°N， 121.4582°E	1283.13	2.39×10^{-6}	Suburban
S2-1	29.9570°N， 121.5352°E	1283.13	4.51×10^{-5}	Suburban
S2-2	29.9582°N， 121.5376°E	1283.13	4.93×10^{-5}	Suburban
S2-3	29.9582°N， 121.5303°E	1283.13	6.07×10^{-5}	Suburban
S3-1	29.9094°N， 121.8380°E	722.11	3.97×10^{-4}	Suburban
S3-2	29.9094°N， 121.8392°E	722.11	4.29×10^{-4}	Suburban
S3-3	29.9094°N， 121.8368°E	722.11	5.00×10^{-4}	Suburban
D1-1	29.8581°N， 121.6768°E	1158.37	4.28×10^{-4}	Developing
D1-2	29.8520°N， 121.6707°E	1158.37	4.22×10^{-4}	Developing
D1-3	29.8581°N， 121.6780°E	1158.37	4.10×10^{-4}	Developing
D2-1	29.8434°N， 121.6182°E	1158.37	5.76×10^{-4}	Developing
D2-2	29.8434°N， 121.6194°E	1158.37	5.22×10^{-4}	Developing
D2-3	29.8446°N， 121.6133°E	1158.37	7.28×10^{-4}	Developing
D3-1	29.8642°N， 121.4448°E	1068.26	9.22×10^{-4}	Developing
D3-2	29.8691°N， 121.4460°E	1068.26	8.70×10^{-4}	Developing
D3-3	29.8678°N， 121.4436°E	1068.26	6.70×10^{-4}	Developing
U1-1	29.8703°N， 121.5608°E	1158.37	3.34×10^{-2}	Urban
U1-2	29.8764°N， 121.5608°E	1158.37	1.19×10^{-2}	Urban
U1-3	29.8788°N， 121.5657°E	1158.37	1.42×10^{-2}	Urban
U2-1	29.8739°N， 121.5498°E	1068.26	3.57×10^{-2}	Urban
U2-2	29.8739°N， 121.5486°E	1068.26	3.12×10^{-2}	Urban
U2-3	29.8727°N， 121.5498°E	1068.26	3.08×10^{-2}	Urban

ALIAN, artificial light intensity at night. Suburban, developing, and urban stand for the corresponding urbanization level for sampling. The data of population density are adapted from Ningbo Statistical Yearbook (2021), and the data of ALIAN are obtained from "Luojia-1" night light remote sensing satellite.

Table S2. The physicochemical properties of the leaf and soil samples plus the light intensity.

	Physicochemical parameter	Suburban	Developing	Urban
Leaf	C (%)	49.44 ± 0.25a	48.75 ± 0.60b	48.02 ± 0.41c
	N (%)	1.94 ± 0.11a	1.61 ± 0.02b	1.29 ± 0.12c
	S (%)	0.14 ± 0.01a	0.12 ± 0.01b	0.11 ± 0.01c
	C/N	25.54 ± 1.63c	30.35 ± 0.71b	37.54 ± 3.33a
	Moisture (%)	0.55 ± 0.02a	0.51 ± 0.01b	0.55 ± 0.02a
Soil	NH_4^+	7.40 ± 5.15ab	3.42 ± 1.09b	9.72 ± 5.35a
	NO_2^-	62.93 ± 49.32a	20.83 ± 12.53a	85.28 ± 80.83a
	NO_3^-	4.19 ± 2.92ab	1.62 ± 2.26b	5.79 ± 2.94a
	pH	7.46 ± 0.33a	6.39 ± 0.86b	6.37 ± 0.32b
Light	ALIAN	$1.65 \pm 2.11\times10^{-4}$b	$6.16 \pm 1.93\times10^{-4}$b	$2.62 \pm 1.04\times10^{-2}$a

ALIAN, artificial light intensity at night. Suburban, developing, and urban stand for the corresponding urbanization level for sampling.

Table S3. PERMANOVA analysis of Bray-Curtis dissimilarities of phyllosphere microbiota of camphor trees along the urbanization gradient.

Variable	F value	R^2	Pr(>F)
Total	1.61	0.13	0.005
Devoloping vs Suburban	3.80	0.19	0.039
Devoloping vs Urban	0.96	0.07	0.481
Suburban vs Urban	4.36	0.25	0.018

Suburban, developing, and urban stand for the corresponding urbanization level for sampling. The P values in the three pairwise adonis analysis results are adjusted P values.

Table S4. Biomarkers screened by LEfSe analysis of phyllosphere microbiota of camphor trees along the urbanization gradient.

Urbanization level	Biomarkernames	LDA Score (log10)	*P*-value
	g__Candidatus	3.32	0.0481*
	g__Labedella	3.61	0.0378*
	g__Microlunatus	3.17	0.0256*
	g__Actinomycetospora	3.61	0.0402*
	g__Atopostipes	3.49	0.0021**
	k__Bacteria	3.78	0.0027**
	f__Koribacteraceae	3.33	0.0481*
Suburban	f__Pseudonocardiaceae	3.62	0.0437*
	o__Solirubrobacterales	3.12	0.0272*
	o__Rhodospirillales	3.42	0.0031**
	f__Acetobacteraceae	3.08	0.0315*
	f__Oxalobacteraceae	4.47	0.0399*
	f__Campylobacteraceae	3.16	0.0403*
	o__Sphaerochaetales	3.23	0.0496*
	f__Sphaerochaetaceae	3.23	0.0496*
	g__Alicyclobacillus	3.50	0.0346*
	g__Enterococcus	3.33	0.0357*
	g__Faecalibacterium	3.12	0.0177*
	g__Brevundimonas	3.91	0.0251*
	g__Methylocapsa	3.56	0.0313*
	g__Desulfocapsa	3.11	0.0307*
	g__Methylosarcina	3.84	0.0295*
	g__Akkermansia	3.41	0.0248*
	k__Archaea	3.78	0.0028**
	p__Crenarchaeota	3.33	0.0153*
	c__Thaumarchaeota	3.33	0.0153*
Developing	p__Euryarchaeota	3.62	0.0201*
	f__Alicyclobacillaceae	3.50	0.0346*
	f__Enterococcaceae	3.35	0.0177*
	f__Thermodesulfovibrionaceae	3.11	0.0410*
	o__Caulobacterales	3.97	0.0251*
	f__Caulobacteraceae	3.97	0.0251*
	f__Beijerinckiaceae	3.64	0.0230*
	f__Rhodospirillaceae	3.32	0.0188*
	c__Verrucomicrobiae	3.45	0.0186*
	o__Verrucomicrobiales	3.45	0.0186*
	f__Verrucomicrobiaceae	3.45	0.0186*
	g__Succiniclasticum	3.31	0.0355*
Urban	g__Albidovulum	3.81	0.0424*
	g__Schlegelella	4.24	0.0345*

	g__Hydrogenophilus	4.43	0.0250*
	g__Desulfovibrio	3.43	0.0270*
	g__Smithella	3.16	0.0026**
	g__Wolinella	3.06	0.0180*
	g__Thalassomonas	3.39	0.0355*
	c__Erysipelotrichi	3.93	0.0213*
	o__Erysipelotrichales	3.93	0.0213*
	f__Erysipelotrichaceae	3.93	0.0213*
	o__Rhodobacterales	3.81	0.0177*
	f__Rhodobacteraceae	3.81	0.0247*
	f__Comamonadaceae	4.41	0.0011**
	o__Rhodocyclales	4.46	0.0289
	f__Rhodocyclaceae	4.46	0.0289*
	o__Alteromonadales	3.62	0.0477*
	f__Colwelliaceae	3.42	0.0355*

Suburban, developing, and urban stand for the corresponding biomarkers of different urbanization levels. An asterisk denotes a significant statistical difference between treatments(*, $p < 0.05$; **, $p < 0.01$). LDA Score (log10), linear discriminant analysis score.

Table S5. The keystone taxa of phyllosphere bacterial networks of camphor trees along the urbanization gradient.

ID	Phylum	Order	Within-module degree (z-score)	Among-module degree (c-score)	Degree	Modularity	Type	Treatment
ASV38	Acidobacteria	Acidobacteria-6	0.59	0.67	64	5	Connectors	suburban
ASV38	Acidobacteria	Acidobacteria-6	-0.32	0.63	13	5	Connectors	developing
ASV41	Acidobacteria	Acidobacteriia	0.61	0.64	54	1	Connectors	suburban
ASV41	Acidobacteria	Acidobacteriia	-2.31	0.67	3	1	Connectors	urban
ASV49	Acidobacteria	Acidobacteriia	0.61	0.66	62	1	Connectors	suburban
ASV49	Acidobacteria	Acidobacteriia	-1.44	0.69	8	3	Connectors	urban
ASV60	Acidobacteria	c_unclassified_p__Acidobacteria	0.74	0.67	15	1	Connectors	urban
ASV95	Actinobacteria	Actinobacteria	0.38	0.66	53	2	Connectors	suburban
ASV101	Actinobacteria	Actinobacteria	-0.60	0.66	8	3	Connectors	developing
ASV107	Actinobacteria	Actinobacteria	0.86	0.65	55	2	Connectors	suburban
ASV202	Actinobacteria	Actinobacteria	0.77	0.65	60	1	Connectors	suburban
ASV202	Actinobacteria	Actinobacteria	-0.66	0.65	12	4	Connectors	developing
ASV214	Actinobacteria	Actinobacteria	1.71	0.64	67	5	Connectors	suburban
ASV222	Actinobacteria	c_unclassified_p__Actinobacteria	-0.02	0.67	46	1	Connectors	suburban
ASV236	Actinobacteria	Thermoleophilia	0.86	0.64	62	2	Connectors	suburban
ASV245	Actinobacteria	Thermoleophilia	-1.57	0.72	6	2	Connectors	developing
ASV252	Armatimonadetes	c_unclassified_p__Armatimonadetes	-0.15	0.65	38	5	Connectors	suburban
ASV266	Bacteroidetes	Bacteroidia	-0.02	0.66	51	1	Connectors	suburban
ASV270	Bacteroidetes	Bacteroidia	0.77	0.66	62	1	Connectors	suburban
ASV270	Bacteroidetes	Bacteroidia	-1.58	0.63	4	1	Connectors	developing
ASV282	Bacteroidetes	Bacteroidia	0.84	0.66	65	5	Connectors	suburban

ASV282	Bacteroidetes	Bacteroidia	-0.60	0.67	6	3	Connectors	developing
ASV376	Chloroflexi	Anaerolineae	-0.98	0.63	8	4	Connectors	developing
ASV381	Chloroflexi	Anaerolineae	0.61	0.66	59	1	Connectors	suburban
ASV391	Chloroflexi	Anaerolineae	0.74	0.63	58	2	Connectors	suburban
ASV395	Chloroflexi	Anaerolineae	0.00	0.70	16	4	Connectors	suburban
ASV398	Chloroflexi	Anaerolineae	-1.29	0.69	7	1	Connectors	urban
ASV402	Chloroflexi	Anaerolineae	-0.28	0.67	46	5	Connectors	suburban
ASV408	Chloroflexi	Anaerolineae	-1.57	0.63	4	2	Connectors	developing
ASV408	Chloroflexi	Anaerolineae	-0.27	0.71	12	1	Connectors	urban
ASV417	Chloroflexi	Chloroflexi	2.58	0.12	16	4	Module hubs	developing
ASV423	Chloroflexi	Ktedonobacteria	0.14	0.66	51	1	Connectors	suburban
ASV423	Chloroflexi	Ktedonobacteria	-0.87	0.65	7	1	Connectors	developing
ASV427	Chloroflexi	Ktedonobacteria	0.61	0.67	55	1	Connectors	suburban
ASV498	Firmicutes	Bacilli	-0.34	0.65	43	1	Connectors	suburban
ASV548	Firmicutes	Bacilli	-0.08	0.64	11	2	Connectors	developing
ASV579	Firmicutes	Clostridia	0.93	0.66	67	1	Connectors	suburban
ASV607	Firmicutes	Clostridia	1.10	0.64	62	2	Connectors	suburban
ASV632	Firmicutes	Clostridia	0.86	0.63	56	2	Connectors	suburban
ASV634	Firmicutes	Clostridia	-0.15	0.64	35	5	Connectors	suburban
ASV728	p_unclassified_k__Bacteria	c_unclassified_k__Bacteria	0.00	0.70	10	3	Connectors	developing
ASV763	Planctomycetes	Planctomycetia	-0.40	0.63	29	5	Connectors	suburban
ASV774	Proteobacteria	Alphaproteobacteria	0.30	0.64	53	1	Connectors	suburban
ASV777	Proteobacteria	Alphaproteobacteria	2.80	0.00	12	2	Module hubs	urban
ASV803	Proteobacteria	Alphaproteobacteria	0.77	0.65	60	1	Connectors	suburban
ASV809	Proteobacteria	Alphaproteobacteria	-1.79	0.67	3	3	Connectors	developing
ASV809	Proteobacteria	Alphaproteobacteria	-2.06	0.63	4	2	Connectors	urban

ASV815	Proteobacteria	Alphaproteobacteria	-1.22	0.70	10	1	Connectors	developing
ASV854	Proteobacteria	Alphaproteobacteria	1.10	0.63	60	2	Connectors	suburban
ASV854	Proteobacteria	Alphaproteobacteria	-1.21	0.63	8	4	Connectors	urban
ASV875	Proteobacteria	Alphaproteobacteria	-0.65	0.66	35	5	Connectors	suburban
ASV893	Proteobacteria	Alphaproteobacteria	1.79	0.67	14	3	Connectors	developing
ASV922	Proteobacteria	Betaproteobacteria	0.47	0.66	51	5	Connectors	suburban
ASV934	Proteobacteria	Betaproteobacteria	0.14	0.67	54	1	Connectors	suburban
ASV938	Proteobacteria	Betaproteobacteria	0.14	0.67	55	1	Connectors	suburban
ASV954	Proteobacteria	Betaproteobacteria	-0.34	0.67	45	1	Connectors	suburban
ASV954	Proteobacteria	Betaproteobacteria	-0.52	0.66	11	1	Connectors	developing
ASV958	Proteobacteria	Betaproteobacteria	-0.60	0.72	9	3	Connectors	developing
ASV998	Proteobacteria	Deltaproteobacteria	0.00	0.63	4	4	Connectors	suburban
ASV999	Proteobacteria	Deltaproteobacteria	0.61	0.67	58	1	Connectors	suburban
ASV1001	Proteobacteria	Deltaproteobacteria	-0.65	0.64	26	5	Connectors	suburban
ASV1018	Proteobacteria	Deltaproteobacteria	-1.07	0.72	9	2	Connectors	developing
ASV1029	Proteobacteria	Deltaproteobacteria	-0.02	0.68	57	1	Connectors	suburban
ASV1067	Proteobacteria	Gammaproteobacteria	-0.66	0.68	11	4	Connectors	developing
ASV1086	Proteobacteria	Gammaproteobacteria	0.93	0.66	62	1	Connectors	suburban
ASV1086	Proteobacteria	Gammaproteobacteria	0.00	0.63	8	3	Connectors	developing
ASV1107	Proteobacteria	Gammaproteobacteria	-0.22	0.68	40	2	Connectors	suburban
ASV1108	Proteobacteria	Gammaproteobacteria	0.77	0.67	59	1	Connectors	suburban
ASV1111	Proteobacteria	Gammaproteobacteria	0.46	0.67	65	1	Connectors	suburban
ASV1112	Proteobacteria	Gammaproteobacteria	0.61	0.64	53	1	Connectors	suburban
ASV1131	Proteobacteria	Gammaproteobacteria	-0.34	0.65	40	1	Connectors	suburban

For modularity, 1, 2, 3, 4 refers to module #1, module #2, module #3, and module #4 respectively, and the other 5 refers to other modules. Threshold value for node network roles: kinless hubs (z-score > 2.5; c-score > 0.62), provincial hubs (z-score > 2.5; c-score ≤ 0.62), connectors (z-score ≤ 2.5; c-score > 0.62) and peripherals (zscore≤ 2.5; c-score ≤ 0.62) were defined according to their within-module degree (z-score) and among-module degree (c-score).

Table S6. Bacterial co-occurrence network of leaf phyllosphere microbiota along the urbanization gradient.

Urbanization level	Node num	Edge num	Positive edge	Negative edge	Average degree	Modularity	Average clustering coefficient
Suburban	228	4763	4400	363	41.78	0.23	0.5982
Devoloping	230	1647	959	688	14.32	0.40	0.3596
Urban	229	1623	872	751	14.17	0.48	0.4613

Suburban, developing, and urban stand for the corresponding co-occurrence network of different urbanization levels. Modularity, degree of nodes tending to differentiate into different network modules. Average clustering coefficient, degree of nodes tending to cluster together.

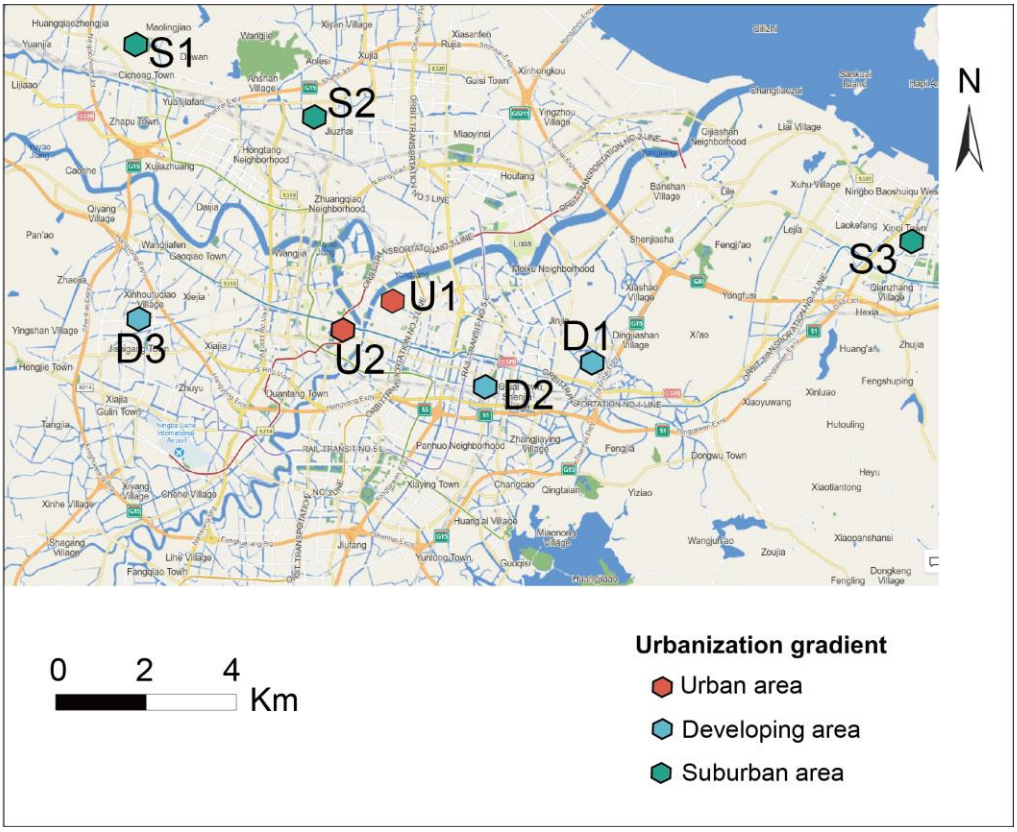

Figure S1. Map of sampling sites for the eight investigated parks along the urbanization gradient.

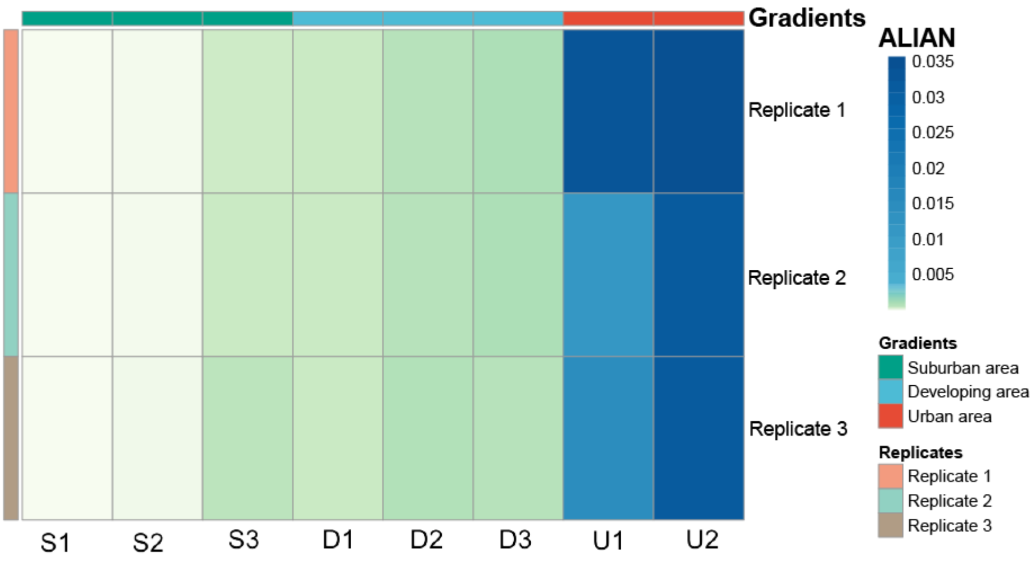

Figure S2. Artificial light intensity at night of 24 sampling sites in 8 parks. Suburban, developing, and urban stand for the corresponding urbanization level for sampling. ALIAN, artificial light intensity at night.

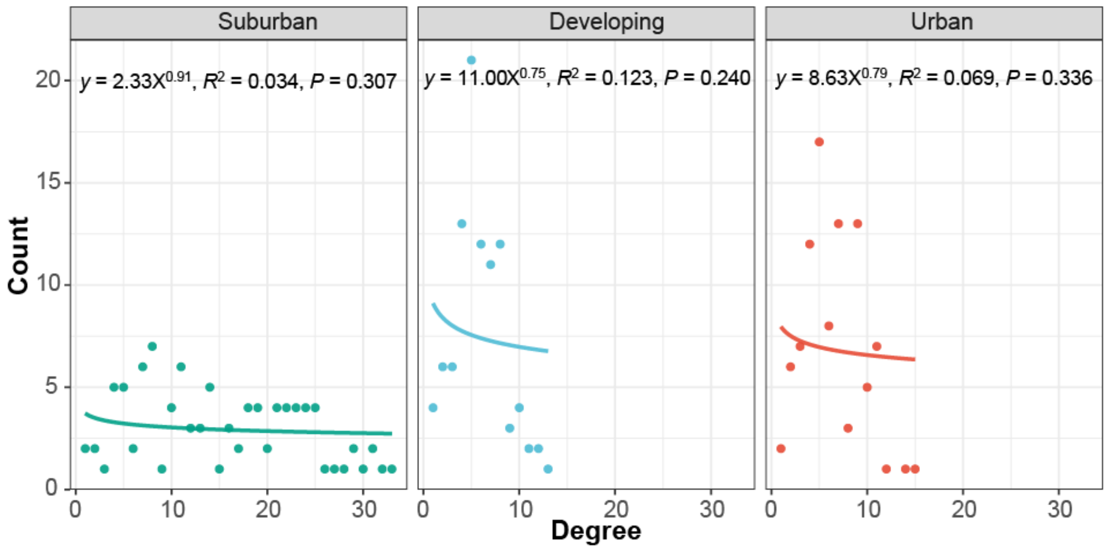

Figure S3. The network degree distribution patterns of phyllosphere microbiota of camphor trees along the urbanization gradient. Suburban, developing, and urban stand for the corresponding co-occurrence network of different urbanization levels.

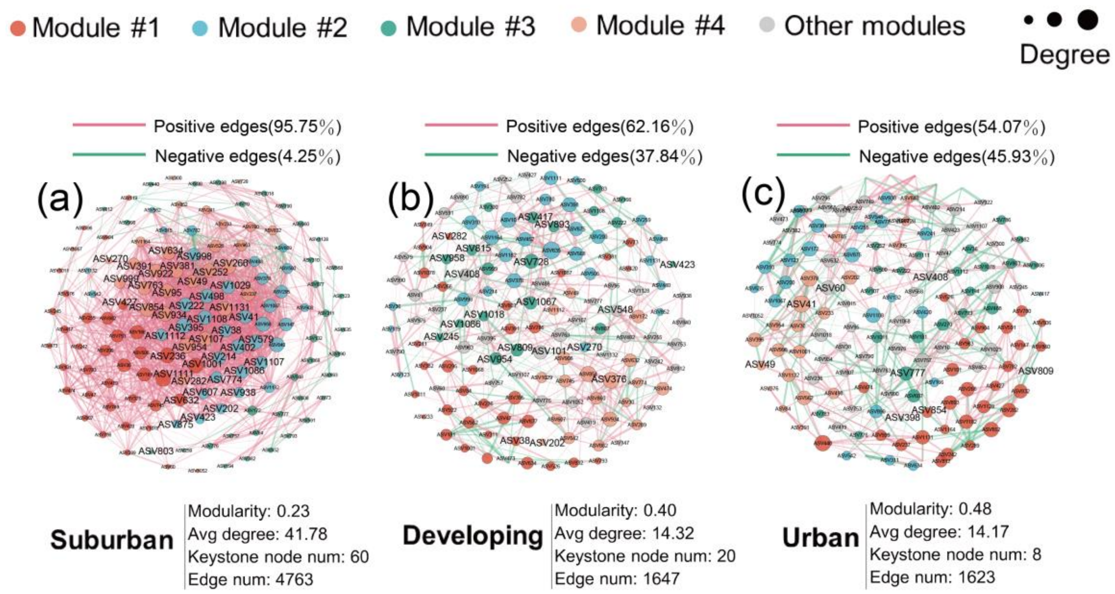

Figure S4. Co-occurrence networks of phyllosphere microbiota of camphor trees along the urbanization gradient. The lines are colored according to positive and negative correlations. Suburban, developing, and urban stand for the corresponding co-occurrence network of different urbanization levels. Avg degree, average degree; Keystone node num, keystone node numbers; Edge num, edge numbers. The node labels in enlarged black font represent keystone species.
